# Research protocol for a digital intervention to reduce stigma among males with a personal experience of suicide in the Australian farming community

**DOI:** 10.1186/s12889-016-3874-3

**Published:** 2016-11-29

**Authors:** Alison J. Kennedy, Vincent Lawrence Versace, Susan A. Brumby

**Affiliations:** 1 National Centre for Farmer Health/Deakin University, Tyers Street, Hamilton, VIC Australia 3300; 2 Deakin University, School of Medicine, Deakin Rural Health (DRH), Princes Highway, Warrnambool, VIC Australia 3280

**Keywords:** Stigma, Suicide, Mental health, Farmer health, Rural health, Digital intervention, Men, Mixed method research, Australia

## Abstract

**Background:**

Australian farming communities have up to twice the suicide rate of the general population. Men, particularly, demonstrate debilitating self- and perceived-stigma associated with an experience of suicide. The Ripple Effect is aimed to reduce suicide stigma within the social, cultural, geographical and psychological contexts in which it occurs.

**Methods:**

A mixed-method design with multi-level evaluation will be effected following the development and delivery of a personalised website experience (combining shared stories, education, personal goal setting and links to resources) to farming men, aged 30–64 years, with an experience of suicide. Pre- and post-surveys will be used to assess changes in self- and perceived-stigma and suicide literacy. Online feedback from participants and semi-structured interviews during follow-up will be thematically analysed.

**Discussion:**

This project will provide information about increasingly accessible, innovative approaches to reducing the debilitating health and wellbeing effects of suicide stigma on a population of Australia’s farmers.

**Trial registration:**

This research protocol was registered with the Australian New Zealand Clinical Trials Registry (ANZCTR) (ACTRN: ACTRN12616000289415) on 7^th^ March, 2016.

## Background

Suicide rates continue to be of concern in Australia, with recent figures of 12.2 per 100,000, of which approximately 75% of deaths were male [1]. Despite similar prevalence of diagnosed mental health conditions in metropolitan and rural areas [2], rural populations are at greater risk of suicide [3]. Within the rural population, farmers—defined by occupation—were identified as dying by suicide at up to twice the rate of the general employed population [4, 5]. The vast majority of farmer suicides were male and aged between 15 and 54 years [5]. These figures do not include individuals not defining themselves as farmers (for example, people with off-farm employment, retired farmers, self-identified ‘farmer’s wives’).

Australia’s male farmers have frequently been identified as conforming to normative behaviours encouraging self-reliance, risk-taking behaviour, a practical solution-focus to problems and an avoidance of emotional vulnerability. This combination leads to an increased risk of suicide, and an increased vulnerability to self-stigma and perceived-stigma when farmers are affected by suicide [6–8].

Suicide stigma is the single strongest correlate with grief difficulties and is associated with ongoing suicidal ideation [9]. Research has clearly identified self-stigma and perceived-stigma among those with a lived experience of suicide, including those bereaved by suicide [10, 11] and those who have attempted suicide [12, 13].

In order to attempt to reduce the stigma of suicide among male farming community members, it is first necessary to understand the socially constructed nature of stigma [14]. The social, cultural and contextual influences on self-stigma and perceived-stigma experienced and expressed by male farming community members must be recognised. As such, this community of interest requires a specifically adapted response [15].

### Impact of self-stigma and perceived-stigma

#### Suicide reporting

Where suicide stigma is prevalent, concealment of cause of death is not uncommon. Where concerns about stigma and confidentiality exist there may also be reduced reporting of suicide [16], particularly given the close social ties within farming communities, where anonymity is low and suicide stigma exists [6, 17].

#### Help seeking

Stigma is one of the most significant barriers to people talking about their suicide ideation and engaging with health services [12, 18]. Self-stigma and perceived-stigma reduces the opportunity to seek and engage professional assistance, particularly if prior contact has been unhelpful or negative [6, 12]. Internationally, men have been identified to experience higher levels of self-stigma and perceived-stigma around psychological problems, particularly those aged between 35–64 years and those who had never received help for psychological problems [19]. Kolves and colleagues [20] described the aversion to help-seeking relative to farming communities as including the stigma reinforced by the traditional masculinist paradigm of farming, the heavy and unrelenting work demands, the lack of access to physical and mental health services and a traditional focus on ‘practical’ problem solving as opposed to seeking help (see also [2, 21–25]). The public stigma associated with seeking psychological help may be internalised as self-stigma and perceived-stigma [26]. This is compounded by the tendency of rural farming community members to delay seeking help until symptoms are so severe as to prohibit one from fulfilling their farming role [27]. For male farming family members impacted by suicide, the need to avoid emotional vulnerability, maintain status and maintain self-reliant patterns of behaviour, reduces access to help even further [7, 28]. Asking for help, particularly for emotional problems, is simply not in the lexicon of many male farming community members and is not recognised within the farming family identity [7]. This is further exacerbated when associated with feelings of weakness, shame, guilt, selfishness and the sense of rejection often associated with the self-stigma and perceived-stigma accompanying a lived experience of suicide [29, 30]. Consequently, those experiencing suicide ideation and those who have attempted suicide are likely to obscure their behaviour [18] and avoid seeking assistance early in the suicidal process [30].

#### Social connection

Self-stigma and perceived-stigma in people with a lived experience of suicide leads to a perception of negative judgement, an avoidance of discussing the experience and their grief with other people [31] and further withdrawal from usual sources of social connection [11], particularly following suicide bereavement [11, 32]. This tendency for social withdrawal further restricts people’s access to the protection from vulnerability that effective social support can provide [18], increasing the potential risk for psychological distress and ultimately the ongoing cycle of suicide. This threat to social interaction and support is more challenging for suicide than other types of loss [33]. Social withdrawal is not only an entrenched passive process; those experiencing self-stigma and perceived-stigma may also actively stimulate avoidance and rejection by others [9]. In rural farming communities, where tightly integrated patterns of working and living are frequent, social disconnection may have a life-altering effect.

#### Ongoing cycle of suicide risk

A lived experience of suicide—whether through suicide ideation, attempted suicide, caring for someone who has attempted suicide, suicide bereavement or being touched by suicide in some other way—significantly increases both the ongoing risk of suicide and threats to mental health and wellbeing [12, 34–36]. Stigma increases the risk of suicide for those already suffering psychologically [19].

### Reducing self-stigma and perceived-stigma

Reducing self-stigma and perceived-stigma associated with a lived experience of suicide has numerous benefits. Firstly, de-stigmatisation improves communication between those with a lived experience of suicide and support networks, thus improving the opportunity for appropriate and acceptable intervention [37]. Secondly, while ingrained, socially constructed factors may restrict help-seeking, reducing self-stigma and perceived-stigma has been described as a way to reduce this barrier [28]. Thirdly, suicide stigma is one of the greatest barriers to generating good quality, accurate data about suicidal behaviour [18]. Reducing self-stigma and perceived-stigma may allow for greater openness from those with lived experience; increasing the knowledge base and enabling the development of appropriate support for those affected. Fourthly, disrupting the negative feedback of self-stigma and perceived-stigma will, over time, lead to a virtuous cycle [38] of reducing the personal and structural stigma of suicide in the broader farming community. Finally, the combination of knowledge, attitude and behaviour change has been suggested as the most effective way to reduce self-stigma and perceived-stigma, particularly through the facilitation of disclosure and the encouragement of positive social contact [38].

### Identifying gaps in stigma reduction research

The funding body for the Ripple Effect—*beyondblue*—identified a number of gaps in stigma research when developing a rationale for the study approach and target sample. These included:

- There was a dearth of evidence about digital interventions as a stigma reduction strategy, despite suggestions that the potential for widespread dissemination through digital communication is likely to play a role in stigma reduction [39].
- There was a lack of programs targeting the needs of people aged 30–64 years, when compared with those for people under the age of 30 years (*beyondblue*, unpublished data, 2013).
- Digital engagement was identified as dropping off after the age of 64 years (*beyondblue*, unpublished data, 2013).
- Perceived-stigma was identified as being higher in men aged 30–64 years (*beyondblue*, unpublished data, 2013).

**Fig. 1:**
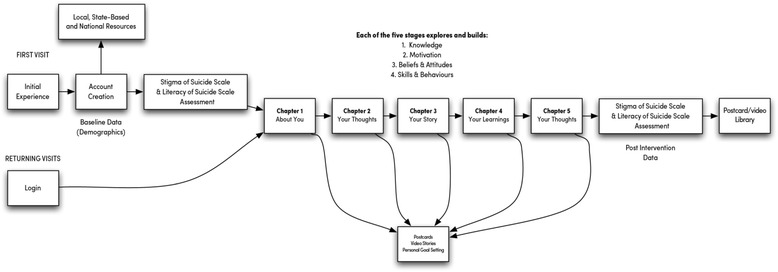
Wireframe of the Ripple Effect intervention

The aim of this protocol is to describe the development, methodology (as outlined in the wireframe in Fig. 1) and proposed evaluation of the Ripple Effect—a digital intervention designed to reduce the self- and perceived-stigma associated with an experience of suicide for men from the Australian farming community aged between 30 and 64 years.

## Methods/design

### Study design

The Ripple Effect encourages participation from a strengths-based perspective working with, rather than against the normative behaviours present in Australian male farmers (e.g. participation as a way of helping your farming community and presenting problems as solvable). Personalised progression through the Ripple Effect allows for targeted and relevant information to increase knowledge, influence attitudes and facilitate behaviour change. Access to additional appropriate support resources is built into the intervention.

The study is designed to include quantitative, qualitative and evaluation components.

- Quantitative
  A non-randomised controlled trial will be used as the quantitative study design. The Ripple Effect website is the intervention and outcomes to be assessed are self-stigma and perceived-stigma about suicide and suicide literacy.
- Qualitative
  The qualitative element of the study design will assess participants’ experience of suicide stigma—including how this influences patterns of help seeking—via short answer questions posed throughout the participation experience.
- Evaluation
  Evaluation of the Ripple Effect will occur:
  At the pilot stage via an online survey and verbal feedback.
  At the post-intervention stage using a 7-point Likert scale, with additional opportunity for including comments.
  At follow-up via semi-structured qualitative interviews.

### Study population

The target population (stipulated by beyondblue, the funding body) are men from the Australian farming community aged 30–64 years who self-identify as having an experience of suicide—bereaved by suicide, attempted suicide, cared for someone who has attempted suicide, had thoughts of taking their own life, or been touched by suicide in some other way. The authors recognise that all members of the rural farming community are likely to be affected by suicide to some degree and believe the Ripple Effect will have benefit beyond the target population. Given this, the primary analysis will focus on men from the farming community with an experience of suicide aged 30–64 years. However, males outside of the target age and females will not be restricted from participating in the Ripple Effect website, and will be included in secondary data analysis. Membership to the farming community will be self-identified. Participants are required to be 18 years of age of over.

### Intervention

The intervention uses a participatory approach to engage with participants informed by Kolb’s [40] experiential learning process and will be guided by a steering group comprising farmers with an experience of suicide, researchers, health professionals, farming industry representatives and digital designers. The intervention will run over a period of 8 months, preceded by a 6-week pilot program.

The Ripple Effect intervention is designed to allow access by as many isolated men (both geographically and psychologically) as possible—irrespective of the type of digital technology (or internet quality) that they have access to in their day-to-day lives. The digital platform delivering the intervention has been optimised for slow connections, and will work equally well on an old Windows PC or a new smart device. The digital platform will allow for both core and curated content to be delivered to participants. These systems will be maintained and scheduled digitally via a highly sophisticated backend. Users (de-identified) can participate anonymously, allowing them to respond in the most genuine and meaningful way possible throughout the process.

The goal of reducing suicide stigma through the Ripple Effect program will be achieved using a combination of different components within the intervention, the website:

1. Shared stories and experiences - participants will have the opportunity to share aspects of the own story as well as read other people's messages (collected via digital Ripple Effect postcards). Postcard messages will be screened, before being digitised and included on the website, to ensure they meet the Mindframe media guidelines [41] for talking about suicide. A series of digital stories—created by members of the farming community with an experience of suicide during a professionally facilitated workshop—will be presented to participants. These stories highlight experiences of suicide stigma and how this can be overcome.
2. Information/education – Participants will be presentation with core and curated information as they move through the website. This information covers five topics as outlined in Table 1. For each of the five topics, all participants will be presented with core content. This core content will take approximately three hours in total (across the combined topic areas) to complete. Additional curated information will be presented during the course of the intervention, depending on the nature of their experience of suicide. For example, participants who identify sexuality as influencing their experience of suicide will be delivered content on suicide risk/protective factors, tipping points and support needs specific to Lesbian, Gay, Bisexual, Transgender and Intersex (LGBTI) people.

**Table 1:** Content of the Ripple Effect

Knowledge about suicide	Everyone’s experience is different	Talking about suicide	Recognising and maximising resources	Knowing what’s needed for keeping well
• Risk/protective factors • Warning signs • Precipitating events • Understanding suicide attempts/thoughts • Suicide stigma	• Cultural and linguistic diversity • Aboriginal and Torres Strait Islanders • Sexuality, sex and gender • Disability, illness and ageing	• Starting and managing conversations with people in distress • Crisis response • Avoiding judgement • Preparation and self-care • Talking in the community about suicide	• Positive and proactive support seeking • Knowing available resources • Overcoming barriers to support • Caring for and supporting others	• Maintaining physical, emotional, intellectual and spiritual health • Personal goal setting (evaluated us behaviourally anchored rating scale – BARS)
3. Personal goal setting – at three set points during the intervention, participants will be invited to set SMART (Specific, Measurable, Agreed, Realistic, Time Specific) personal goals with the aim of reducing stigma. Participants will be asked to report back (within two weeks of setting the goal) on their achievement of these goals using a behaviourally anchored rating scale (BARS).
4. Resources/support services - participants will be provided with an extensive list of resources and social and emotional wellbeing support services with national, state and local availability upon registration with the Ripple Effect.

### Study tools

#### Quantitative

The quantitative tools for assessing self-stigma and perceived-stigma have been adapted from the short form of the Stigma of Suicide Scale (SOSS) [42]. The introductory statement for the SOSS was adapted—in collaboration with the lead SOSS author [43] to focus on self-stigma and perceived-stigma. Suicide literacy was assessed using the Literacy of Suicide Scale (LOSS) [44]. The SOSS [42] and LOSS [44] have robust psychometric properties and have been validated for Australian community samples. The SOSS [42] is the only suicide attitude scale that focuses specifically on measuring stigma.

#### Qualitative

Personal insights of where stigma was/was not experienced and how stigma was overcome are measured using qualitative tools including a) questions exploring nature of suicide experience, access to support and willingness to talk about suicide; b) opportunity to complete unstructured, online ‘postcards’ of personal insights about an experience of suicide; and, c) opportunity to set unstructured personal goals and report back on these goals 7–10 days later.

#### Evaluation

Tools for evaluation will be administered in three stages (as described in Table 2):

**Table 2:** Evaluation timeline for the Ripple Effect

Ripple Effect Evaluation measures	Pre-intervention	During intervention	Post-intervention
Demographics			
• Age	✓		
• Gender	✓		
• Location (postal code)	✓		
• Farming type	✓		
Detail of suicide experience		✓	
Suicide stigma (SOSS) [42]			
• Self-stigma	✓		✓
• Perceived-stigma	✓		✓
Suicide literacy (LOSS) [44]	✓		✓
Personal goal achievement		✓	✓
Participant feedback			✓
Qualitative interviews			✓

- The initial stage of process evaluation will involve a pilot implementation of the Ripple Effect with members of the steering group. These members collectively provide extensive experience of the farming context and clinical mental health experience, and have knowledge about the aims of the Ripple Effect. Pilot participants will provide extensive online feedback via a qualitative survey as well as contribute to a group teleconference for further discussion of feedback that will be used to inform the refinement of the Ripple Effect.
- Following the completion of post-intervention quantitative tools (SOSS and LOSS), online feedback will be sought from the participants by way of a quantitative survey using a 7-point Likert scale, with opportunity for qualitative written comment.
- Follow-up semi-structured qualitative interviews with a small number of participants will investigate a) the further impact of the Ripple Effect on self-stigma and perceived-stigma, complementing and adding richness to the quantitative data in order to more clearly define what is helpful in reducing suicide stigma [12], and b) the experience of participating in the Ripple Effect.

### Data collection

Data collection will be via de-identified individualised digital interaction with participants, mapping their pattern of participation and change across time. Upon registration and prior to exposure to the intervention, participants will complete the Stigma of Suicide Scale - Short Form (SOSS) [42]—adapted to measure self-and perceived-stigma—and the Literacy of Suicide Scale (LOSS) [44].

The Ripple Effect intervention will be undertaken flexibly—participants can self-pace over a maximum 12-week period to complete the five core components. Participants are able to move in and out of the intervention as it suits them. The nature of the registration system allows for people to pick up where they left off. A personal profile page allows participants to identify their progress through the intervention. Participants will receive an email/SMS reminder if they have not logged in to the Ripple Effect after a certain period of time (determined by the participant).

On completion of all of the core content, participants will again complete the SOSS and LOSS tools (post-test). Participants who do not complete all core units will be sent an electronic reminder encouraging them to complete the post-test. Completing participants (all core content of the intervention) will be invited to participate in a semi-structured follow-up interview about their experience of participation in the Ripple Effect. A researcher with doctoral-level experience in interviewing farmers with an experience of suicide will conduct the interviews.

### Sample size

The sample size was based upon the difference in the score of the SOSS tool (pre and post-intervention). Based upon a small effect size (d = 0.2), power of 0.80 and significance level of 5% (α = 0.05), and allowing for 20% attrition, we will aim to recruit 473 participants. As was done by Taylor-Rodgers and Batterham (2014), the power calculation is conservative as it does not account for repeated measures. Purposive sampling will be used to identify 10 participants willing to contribute to the follow-up interviews.

### Data analysis

The SOSS short form (primary outcome measure) and LOSS (secondary outcome measure) will be administered at the beginning, and following completion of the Ripple Effect as an exit survey. The comparison of pre and post data will indicate changes of both stigma and literacy variables of participants in response to the intervention. Analyses will be performed in SPSS version 23 or later. The full analysis set (FAS) will be used adhering to the intention to treat principle (ITT). This will be complemented with analysis of a per protocol set (PPS) that only includes those who have completed the core components (defined as ‘completers’—participants who have worked their way through the core content and completed pre and post measurement of stigma and literacy). Comparing the ITT with the PPS will provide a sensitivity analysis for both the SOSS and LOSS. Paired t-tests will be used to assess the effect of the intervention over time (pre- and post-Ripple Effect). In the event some participants have missing data for the post intervention assessment of SOSS and LOSS, t-tests will be replaced by a mixed model analysis using the method of residual (or restricted) maximum likelihood (REML). Supplementary analyses will include measuring changes in the SOSS and LOSS in relation to the exposure to the intervention, with exposure defined as number of core units completed. Two-sided tests will be used with a level of *p* < 0.05 determining statistical significance.

Interview data will be thematically analysed based on indications of stigma reduction, and changes in help seeking behaviours and knowledge.

### Outcomes

The Ripple Effect’s outcomes will include:

- Learning and reporting about what works to reduce self-stigma and perceived-stigma associated with an experience of suicide within the farming community (as measured by the SOSS [42]).
- Learning and reporting about what works to increase literacy of suicide within the community of farming (as measured by the LOSS [44]).
- Strengthening participants’ self-perception to enable them to assist others who are suffering, thereby reducing the self-perception of shame and isolation often associated with an experience of suicide.
- Increasing the knowledge base of suicide and its experience, and the experience of associated stigma, in rural farming communities.
- Increasing the knowledge of appropriate and acceptable ways to deliver social and emotional wellbeing messaging to members of the farming community.

## Discussion

Digital media is becoming a powerful instrument for rural people in breaking down barriers of rural isolation, allowing for engagement and social interaction. The Ripple Effect utilises these opportunities to aid those with a lived experience of suicide, while also filling gaps in knowledge on the most effective experiences and processes to meaningfully shift the self-stigma and perceived-stigma associated with suicide. It is anticipated that the approach used for this study will also improve literacy of suicide in the target community. The intervention will serve as a tool to assess the extent to which digital content can alter the perceptions and feelings of individuals, with opportunities to refine and reiterate the content to further hone the most effective experiences and techniques to reduce stigma. The intervention will collect numerous data from those who use it, providing insight into the age, location and gender of all users, which content they access and how their perceptions shift. While this project is specifically focused on men aged 30-64 years, it will also be possible to track: a) men outside of this target age, and b) to what extent women use the intervention as a means of supporting their own families or partners facing a lived experience of suicide and the associated stigma. The Ripple Effect will contribute to the burgeoning knowledge of suicide stigma and literacy collected respectively by the SOSS [42] and LOSS [44] to date. Importantly, it will provide new and important knowledge about the experience of Australia’s community of farming, who have a high level of lived experience of suicide. This knowledge will inform policy, service delivery and health promotion strategies in order to more effectively support the wellbeing of rural farming communities.

### Limitations and strengths

The very nature of a digital intervention raises some study limitations. In rural areas particularly, online connectivity can be slow and of poor quality. The Ripple Effect has been designed to respond to these challenges by incorporating bandwidth optimisation for viewing images and video content. Text transcripts can also be accessed when video cannot be accessed and for participants who may be gearing impaired. Restricted access to technology will also be a potential limitation. In response to this, the Ripple Effect has been designed with optimal access from a wide range of devices—from an older desktop computer to a smart phone or tablet—to make the website accessible to as wide a range of potential participants as possible. Findings will be limited, however, to people who have access to some form of digital technology.

The farming focus of the Ripple Effect is both a limitation and a strength of the intervention. The results of this research will not be generalisable beyond the rural farming community (although may have some bearing on rural farming communities in similar contexts internationally, e.g. Canada), despite adding to the knowledge about suicide stigma and literacy in a yet unstudied population. However, this strong focus on farming provides a level of personalisation, cultural familiarity and accessibility that would not be possible in a less tailored intervention.
